# Supplementary figures and images for: Evaluation of photoreceptor features in retinitis pigmentosa with cystoid macular edema by using an adaptive optics fundus camera
Source: PLoS One. 2024 Jan 2;19(1):e0296493. doi: 10.1371/journal.pone.0296493 (PMC10760661; doi:10.1371/journal.pone.0296493)

(a) Total  
(N=54)

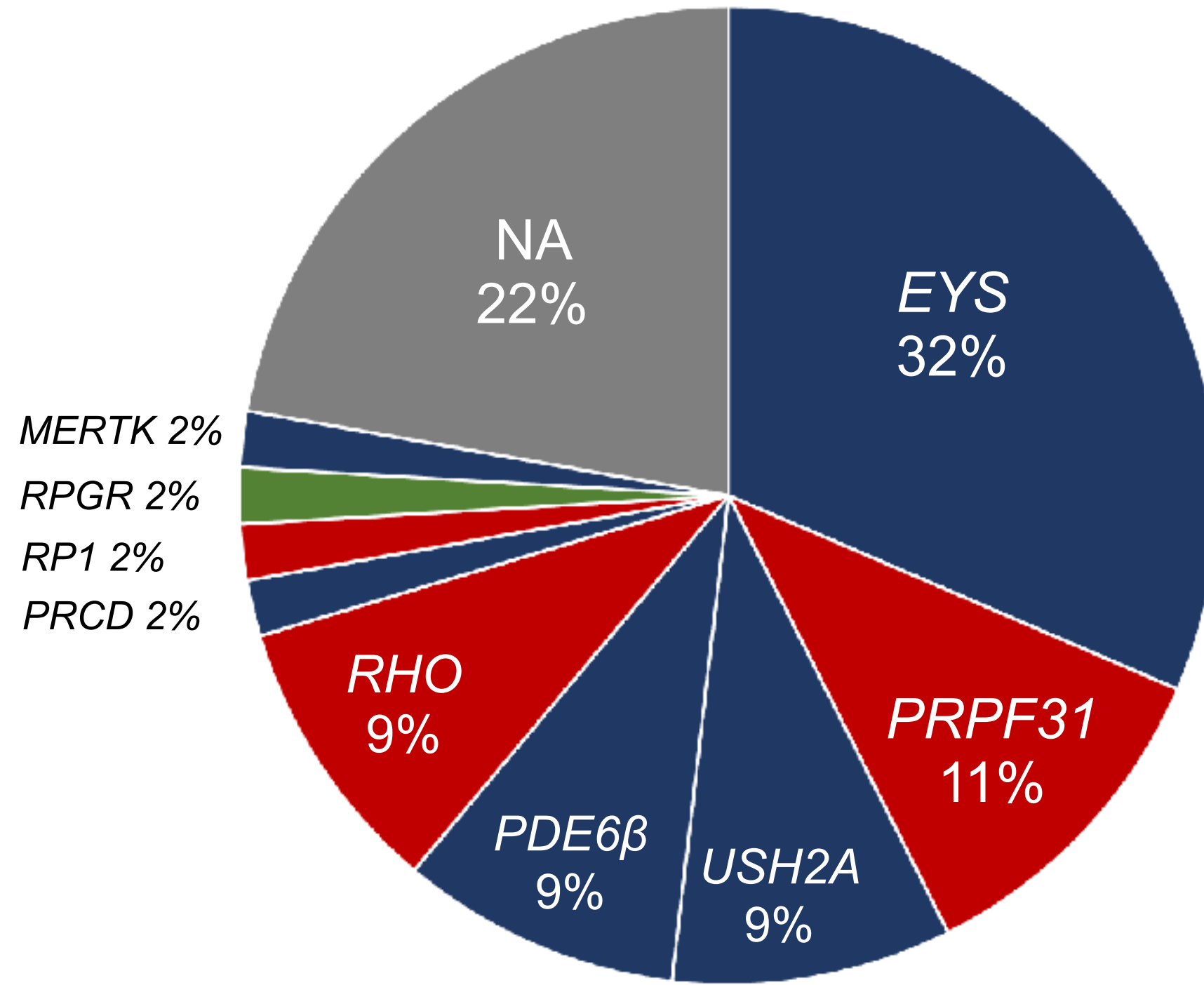

(b) CME Positive  
(N=30)

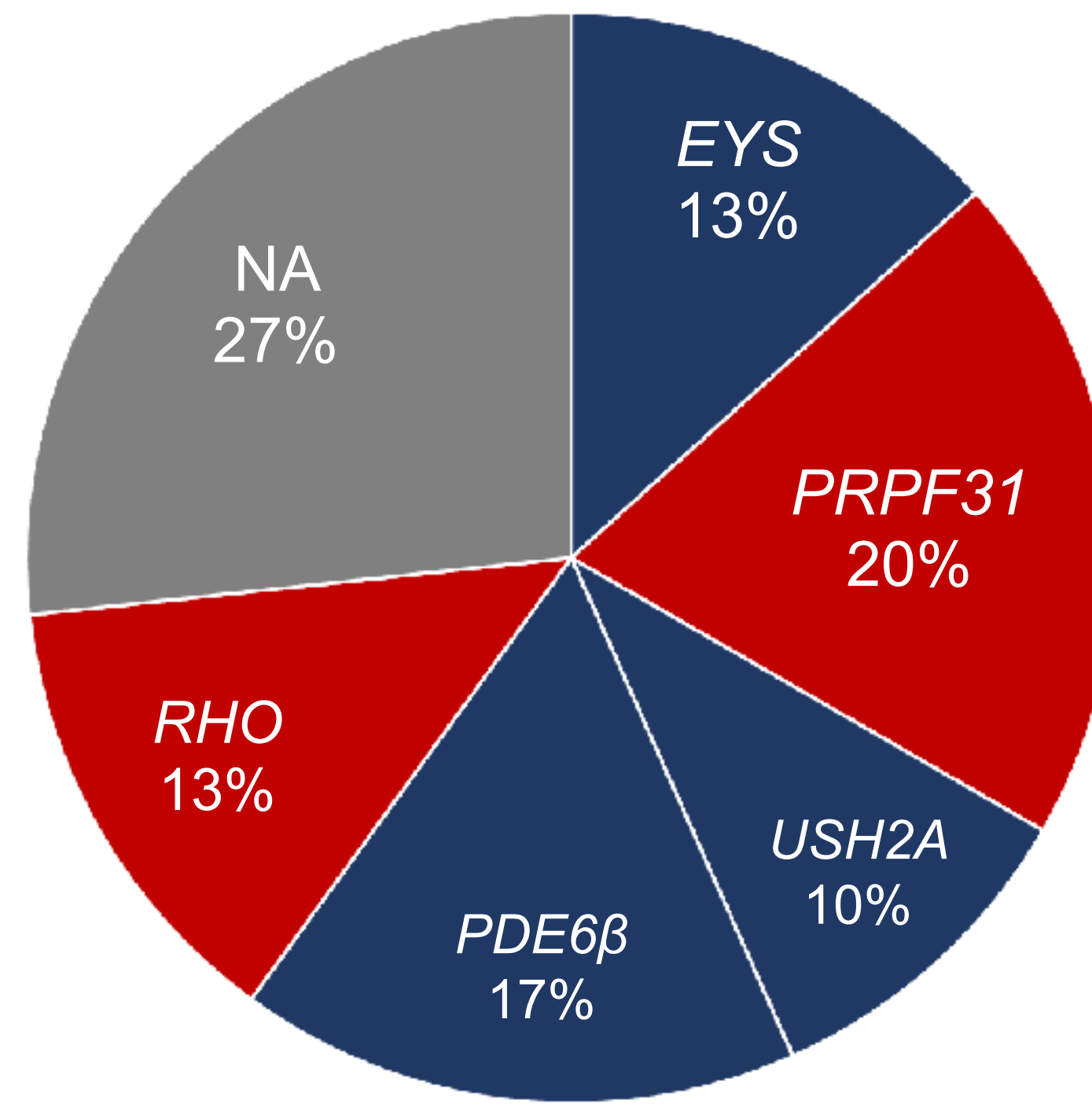

(c) CME Negative  
(N=24)

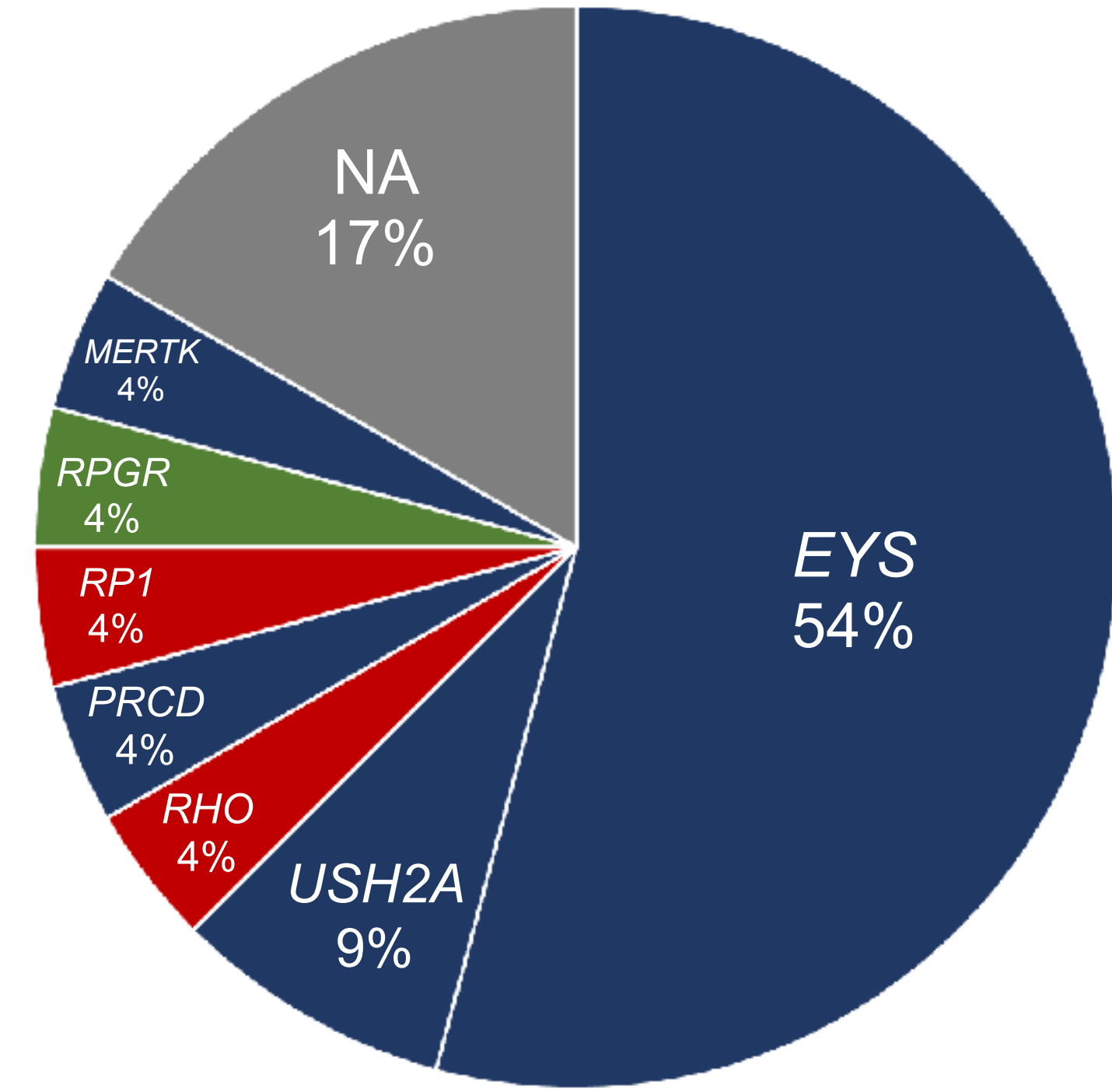

■ Autosomal Recessive

■ Autosomal Dominant

■ X-linked

Supplement: S1 Fig — (a) A total of 54 eyes of 42 patients. (b) CME-positive group consisting of 30 eyes of 18 patients. (c) CME-negative group consisting of 24 eyes of 24 patients. (PDF) [file pone.0296493.s001.pdf]

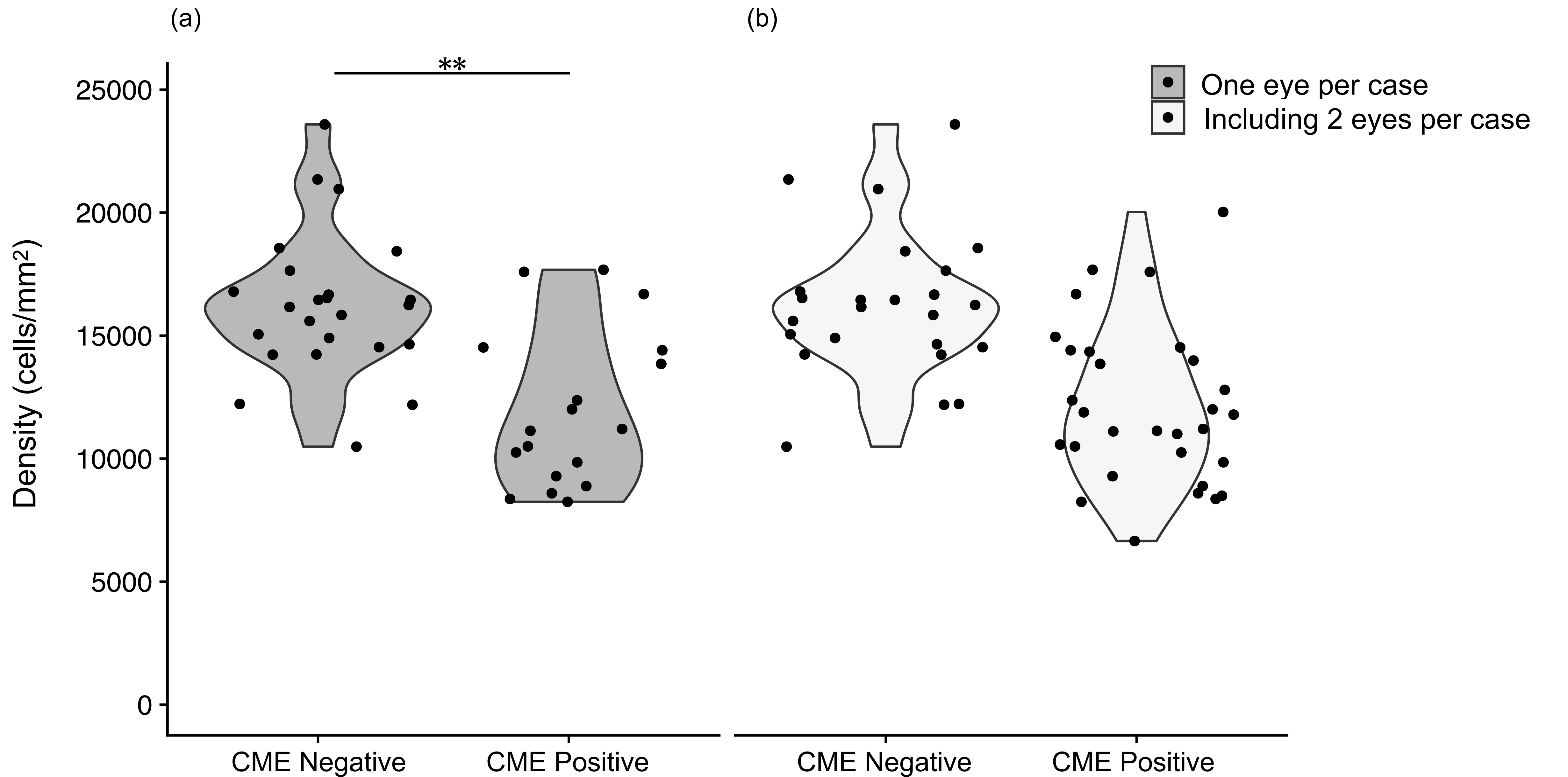

(t-test; \*\*p < 0.01)

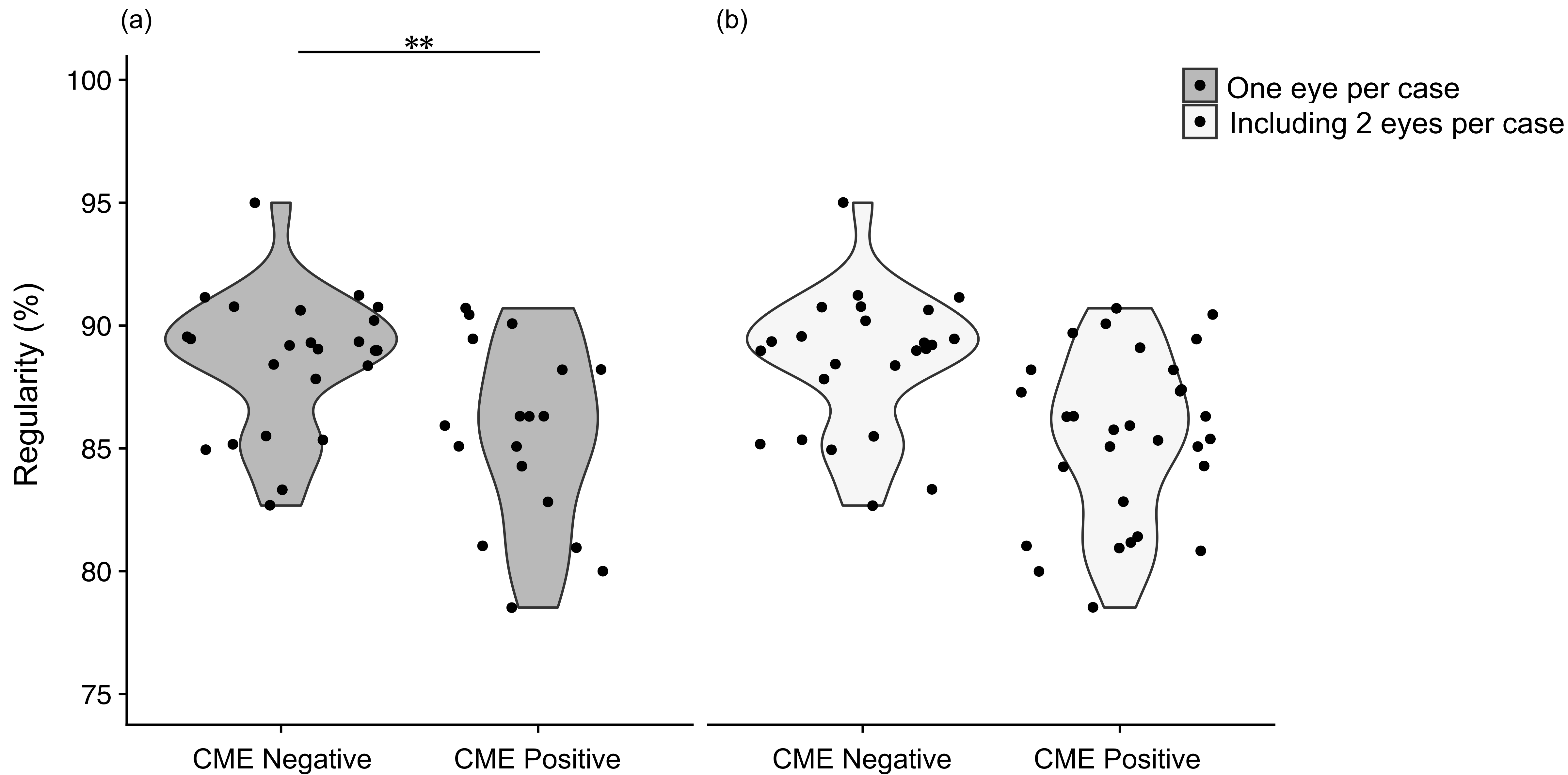

(t-test; \*\*p < 0.01)

Supplement: S2 Fig — (a) One eye per case. (b) Two eyes per case. (PDF) [file pone.0296493.s002.pdf]

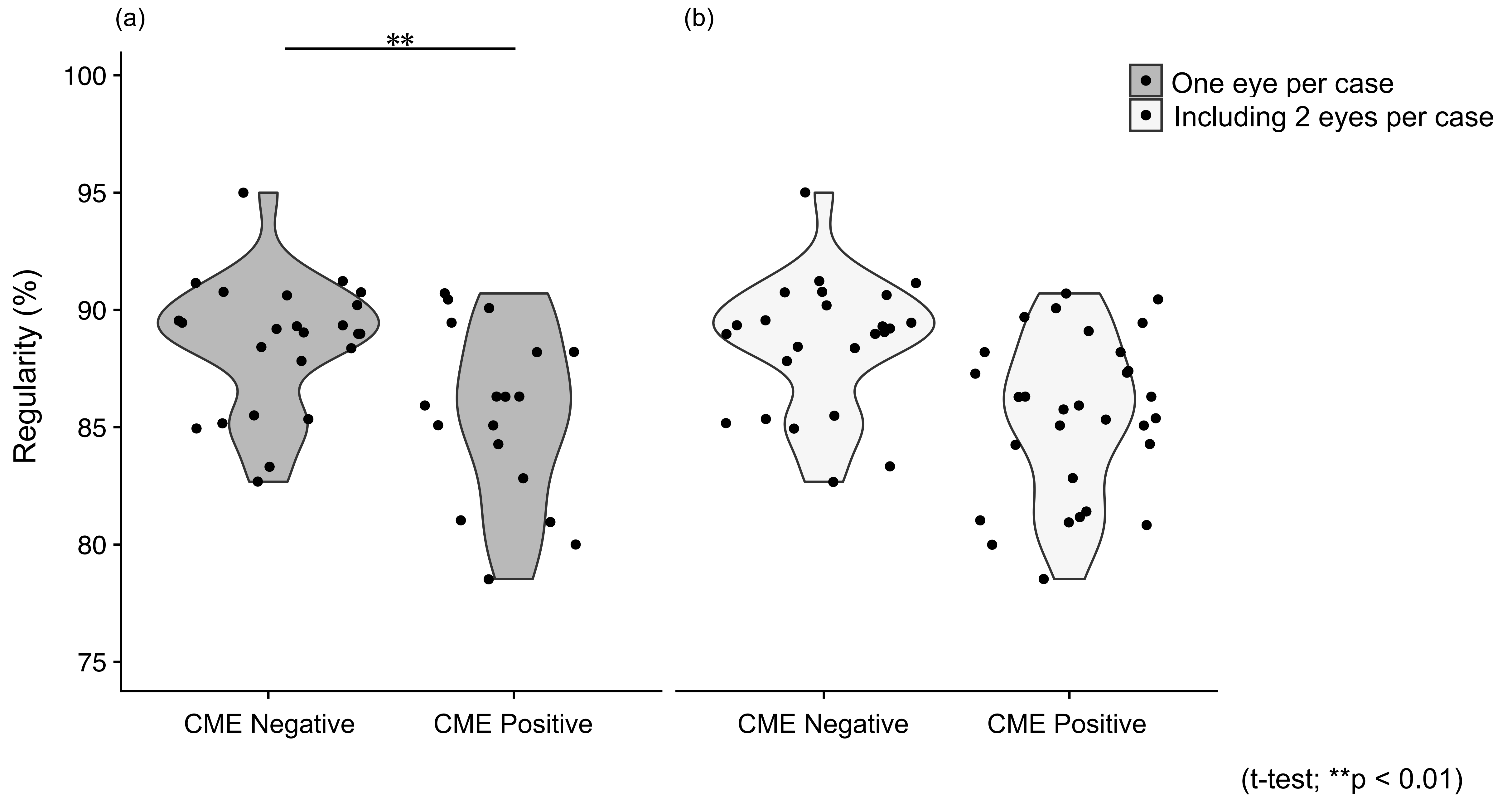

Supplement: S3 Fig — (a) One eye per case. (b) Two eyes per case. (PDF) [file pone.0296493.s003.pdf]
