# Supplementary material for: Evaluation of photoreceptor features in retinitis pigmentosa with cystoid macular edema by using an adaptive optics fundus camera
Source: PLoS One. 2024 Jan 2;19(1):e0296493. doi: 10.1371/journal.pone.0296493 (PMC10760661; doi:10.1371/journal.pone.0296493)
Supplement: S1 Table — (DOCX) [file pone.0296493.s004.docx]

Supplemental Table 1. List of gene mutations associated with retinitis pigmentosa

| Pt. No | Causative gene | Nucleotide change | Protein change | Zygosity | Classification |
| --- | --- | --- | --- | --- | --- |
| 1 | EYS | c.2528G>A | p.Gly843Glu | homo | likely pathogenic |
| 2 | EYS | c.8868C>A  c.4957dupA | p.Tyr2956*  p.Ser1653fs | hetero | pathogenic  pathogenic |
| 3 | MERTK | c.225delA | p.Gly76fs | homo | pathogenic |
| 4 | EYS | c.4957dupA  c.2528G>A | p.Ser1653fs  p.Gly843Glu | hetero | pathogenic  likely pathogenic |
| 5 | None |  |  |  |  |
| 6 | EYS | c.2528G>A | p.Gly843Glu | homo | likely pathogenic |
| 7 | EYS | c.141A>T  c.4957dupA | p.E47D  p.Ser1653Lys fs*2 | hetero  homo | likely pathogenic  pathogenic |
| 8 | EYS | c.8868C>A  c.4957dupA | p.Tyr2956*  p.Ser1653fs | hetero | pathogenic  pathogenic |
| 9 | None |  |  |  |  |
| 10 | USH2A | c.13010C>T  c.10353_10356delTCAT | p.Thr4337Met  p.His3452fs | hetero | pathogenic  pathogenic |
| 11 | PRCD | c.2T>C |  | homo | pathogenic |
| 12 | RPGR | c.281G>A | p.Cys94Tyr | hemi | likely pathogenic |
| 13 | EYS | c.4957dupA  c.2528G>A | p.Ser1653fs  p.Gly843Glu | hetero | pathogenic  likely pathogenic |
| 14 | EYS | c.8868C>A | p.Tyr2956* | homo | pathogenic |
| 15 | EYS | c.8196_8200delCTTTC  c.2528G>A | p.Phe2733fs  p.Gly843Glu | hetero | likely pathogenic  likely pathogenic |
| 16 | EYS | c.4957dupA  c.2528G>A | p.Ser1653fs  p.Gly843Glu | hetero | pathogenic  likely pathogenic |
| 17 | EYS | c.4957dupA  c.1211dupA | p.Ser1653fs  p.Asn404fs | hetero | pathogenic  pathogenic |
| 18 | EYS | c.2528G>A | p.Gly843Glu | homo | likely pathogenic |
| 19 | EYS | c.4957dupA  c.2528G>A | p.Ser1653fs  p.Gly843Glu | hetero | pathogenic  likely pathogenic |
| 20 | None |  |  |  |  |
| 21 | RHO | c.50C>T | p.Thr17Met | hetero | pathogenic |
| 22 | RP1 | c.5797C>T | p.Arg1933* | homo | likely pathogenic |
| 23 | USH2A | c.11815G>T  c.2802T>G | p.Glu3939*  p.Cys934Trp | hetero | pathogenic  pathogenic |
| 24 | None |  |  |  |  |
| 25 | EYS | c.8868C>A | p.Tyr2956 | homo | pathogenic |
| 26 | RHO | c.568G>T | p.Asp190Tyr | hetero | pathogenic |
| 27 | None |  |  |  |  |
| 28 | None |  |  |  |  |
| 29 | EYS | c.8868C>A | p.Tyr2956 | homo | pathogenic |
| 30 | USH2A | c.11811_11812delCT  c.2802T>G | p.Tyr3938fs  p.Cys934Trp | hetero | likely pathogenic  likely pathogenic |
| 31 | RHO | exon5：c.1005delT | p.Thr336fs | hetero | likely pathogenic |
| 32 | PRPF31 | entire deletion |  | hetero | pathogenic |
| 33 | USH2A | c.5608C>T  c.12305T>A | p.Arg1870Trp  p.Ile4102Asn | hetero | likely pathogenic  VUS |
| 34 | EYS | c.7919G>A  ex6-8 del | p.Trp2640 | hetero | pathogenic  likely pathogenic |
| 35 | PDE6β | c.1604T>A  c.1712C>T | p.Ile535Asn  p.Thr571Met | hetero | likely pathogenic  likely pathogenic |
| 36 | PDE6β | c.1669C>T  exon 2-3 del | p.His557Tyr | hetero | likely pathogenic  likely pathogenic |
| 37 | None |  |  |  |  |
| 38 | PRPF31 | ex2 ex3 deletion |  | hetero | pathogenic |
| 39 | PDE6β | c.1604T>A  c.1712C>T | p.Ile535Asn  p.Thr571Met | hetero | likely pathogenic  likely pathogenic |
| 40 | PRPF31 | ex2_3 deletion |  | hetero | pathogenic |
| 41 | None |  |  |  |  |
| 42 | PRPF31 | c.527+1G>A |  | hetero | pathogenic |
