# Supplementary material for: Evaluation of photoreceptor features in retinitis pigmentosa with cystoid macular edema by using an adaptive optics fundus camera
Source: PLoS One. 2024 Jan 2;19(1):e0296493. doi: 10.1371/journal.pone.0296493 (PMC10760661; doi:10.1371/journal.pone.0296493)
Supplement: Attachment — Submitted filename: Response to Reviewers.docx [file pone.0296493.s005.docx]

Response to Reviewers

Evaluation of Photoreceptor Features in Retinitis Pigmentosa with Cystoid Macular Edema by Using an Adaptive Optics Fundus Camera (Manuscript #; PONE-D-23-24372)

All Concerned,

Thank you for inviting us to submit a revised manuscript. We appreciate the time and effort dedicated to providing insightful feedback to strengthen our manuscript. We have worked to address each concern by additional analysis and revising the text for clarity and added results. Thus, with great pleasure we submit our revised manuscript for further consideration. We have incorporated changes that reflect the detailed suggestions you have graciously provided. Hereafter we provide direct responses to each concern raised by the referees.

**Reviewer #1**

**The authors used AO-SLO to evaluate photoreceptors in RP-associated CME. CME is a relatively common complication in RP, and investigating the etiology is clinically important. However, there are some concerns for the interpretation of the result. Specific comments are listed below.**

Thank you for your encouraging comments. Our study was conducted using the rtx1, an AO fundus camera. Although some of the resolution may be degraded due to the fundus camera, we took advantage of the distortion free images.

**1. Line 93, All participants provided written informed consent. However, the study is retrospective and the written informed consent seems not mandatory. In addition, the study was permitted in 2020, and the data between 2015 and 2021 were collected. Was the informed consent obtained for another study such as genetic testing? Please specify.**

Patients underwent examinations from 2015 to 2021 and were later analyzed retrospectively. Even though informed consent wasn't strictly necessary for this retrospective study, we still obtained informed consent from all patients for genetic testing and included it in our research. We have revised the statement for greater specificity. (Line 83).

**2. Line 95 visual field constriction not construction**

We corrected this text. (Line 90)

**3. Line 151, Although the authors state that the interpretation is based on ACMG guidelines, the presented criteria seem not follow the guideline. Specifically, null variant can be categorized as PVS1 but PVS1 without a strong or moderate category is considered VUS. Allele frequency of 5% is too high as a cut-off value. Even if the cut-off were accepted, the assigned category is PM2 and requires PVS, PS, or another 2 PM category to be pathogenic/likely pathogenic. Positive results of in-silico analysis are PP3 and need even more evidence.**

Thank you for pointing this out. We have followed your advice and rewritten the text. Also, related to comment 4, we have attached a new table about gene variants.

**4. Please specify the identified variants in addition to gene names.**

As mentioned in our response to comment 3, we have attached a new table of genetic mutations as a supplemental table 1.

**5. Line 223, The authors did not find a positive correlation between EZ/IZ length and photoreceptor cell counts in CME-positive group. However, the p-value is 0.10, and the non-significance may be due to insufficient sample size. Discussing the difference between significant and non-significant p-value of the comparison should be avoided.**

As you pointed out, there was a significant difference, but it is undeniable that the number of cases is small. We rewrote the sentence from "Thus, the reduction in photoreceptors in the CME-positive group, regardless of EZ/IZ preservation, indicates the relevance of CME.” to “Although it is difficult to be sure because of the small number of patients, the reduction in photoreceptor density in the CME-positive group, regardless of EZ/IZ preservation, might be due to CME.” (line 276).

**6. Line 312, In contrast, the authors discussed CME may disrupt EZ/IZ structure and impair retinal function despite that there was no significant difference in EZ/IZ between CME positive and negative groups (Table 3). Visual acuity looks even better in the CME-positive group. The authors should reconsider the interpretation of the results.**

There were no significant differences in the length of the EZ/IZ, whether CME was positive or negative. This suggests that there was no difference in RP progression in both groups, and visual acuity didn't differ significantly either. As observed in patients with macular telangiectasia type 2[1], photoreceptor density decreased even when the EZ remained intact. In essence, EZ/IZ length alone doesn't accurately reflect photoreceptor density. An important finding of this study is that, even if there wasn't a significant difference in IZ/EZ length, there was a difference in photoreceptor density. In conclusion, our results highlight that only AO examination revealed a significant difference in photoreceptor density between the CME-positive and CME-negative groups. Therefore, we emphasize the importance of using AO examination to assess photoreceptor density.

**7. Related to the above comment, the question is whether the CME-negative group is an appropriate control for the CME-positive group. How did the authors select the CME-negative cases?**

Regardless of the CME-positive or negative, there was a significant reduction in photoreceptor density in the group of patients with RP when compared to a previously reported normal group[2]. Moreover, when we compared RP patients with and without CME complications, we found that photoreceptor density was notably lower in the CME-positive group. The main goal of this study was to understand how CME affects photoreceptor density in RP patients. To do this, we compared patients with and without CME over a specific period of time. We have rewritten it to make it easier to understand in discussion section (line 251).

**8. Is it possible that the presence of CME affects the AO-SLO imaging and results in lower cell density?**

For each photoreceptor analysis examination in AO, we captured the scans of the four perifoveal areas of the retina, as the regions of interest (ROI): superior, inferior, temporal, and nasal, 1.5° to 2° from the center of the fovea. The data from the four ROIs were averaged and used for all analyses in this study. Considering the resolution of AO images, measurements were obtained from regions in which CME had occurred previously but not in areas with current edema. Therefore, AO measurements were taken to avoid the effects of CME. We have rewritten it in methods section. (line 127)

**9. As the authors introduced, studies on AO-SLO findings in RP is limited. Nevertheless, the citation list seems not comprehensive. Previous studies on RP would be more relevant to the present study than MacTel type 2 or GA papers.**

As far as we tried, could not find any reports of photoreceptor density studies using AO in RP patients with CME. Although it has been known that CME is one of major complications of RP, no AO analysis has been performed to date. This is why we conducted this study.

**Reviewer #2: In this study, the authors evaluated the photoreceptor features in retinitis pigmentosa with cystoid macular edema using an adaptive optics fundus camera. They use many Tables and Figures for various analysis, but I recommend that they need to be clear and compact this text, tables, and figures.**

Thank you for your very helpful advice. We have removed the description related to regularity because the important point we wanted to make in this study was about photoreceptor density. We have also removed the figure and table to make them more compact. We think it is now easier to understand.

**1. In this study, CME-positive group include the 13 eyes in current and the 5 eyes in previous. Does the presence of CME affect the photoreceptor mosaic imaging due to adaptive optics fundus camera image quality issues? Although the number of cases is small, is there any difference between the 13 eyes with current CME and the 5 eyes with previous CME?**

As you noted, it was difficult to get accurate measurements with the AO camera in areas where edema was occurring. Therefore, in this study, the edema area was avoided and the examination was performed within the range where the AO camera could take images. There was not significant difference in the AO test results between those with current edema and those with a history of edema. However, the points you have raised are worthy of attention, and we would like to increase the number of cases in the future.

**2. In this data, the integrity of the ellipsoid zone and interdigitation zone is not significantly different between CME-positive group and CME-negative group, but there is a significant difference in photoreceptor density and regularity of AO-camera. Visual acuity is also not significantly different between the two groups, so could this reduction in photoreceptor density and regularity just not reflect well due to the presence of CME? Is there any study that shows that CME causes faster progression of retinitis pigmentosa or faster loss of central vision? The authors need to be considered more.**

It is known that the IZ/EZ shortens as RP progresses, so the fact that there was no significant difference in the length of the EZ/IZ between the presence and absence of CME in this study suggests that the progression of RP is similar. Although there was no significant difference in visual acuity, previous reports have reported that CME complications in RP are not necessarily correlated with loss of visual acuity[3]. Since there were no significant differences in other factors other than CME complications in this study, such as age, EZ/IZ length, and visual acuity, we believe that there was a decrease in photoreceptor density due to a presence and a history of CME, as has been noted in other diseases[1,4,5]. In the group with at least once CME complication existed, there was a negative correlation between density and age distribution. On the other hand, there was no correlation with age in the CME-negative group. We therefore suggest that CME treatment should be given as early as possible.

**3. Figure 5-7: Please note the P-values and correlation coefficients in the figure or figure legend.**

Thank you for pointing this out. It would certainly make it easier to understand that way. We have added and modified it accordingly.

**4. Table 4-5: Please clearly indicate in the table title that the data is only for the CME-positive group.**

We have added “in the CME-positive group” to the table title.

**5. Table 4-5: How did the authors define disease duration?**

Since SD-OCT examination was always performed at each consultation visit of all patients in our hospital. The duration of CME was decided by the period from the first point of the edema was diagnosed until the last visit it was positive in the SD-OCT. When CME observed in the previous visit showed improvement in the next outpatient visit, the duration was defined until the previous visit. We added it to the text.

**Reference**

1.	Jacob J, Krivosic V, Paques M, Tadayoni R, Gaudric A. Cone density loss on adaptive optics in early macular telangiectasia type 2. Invest Ophthalmol Vis Sci. 2015;56: 90–90.

2.	Legras R, Gaudric A, Woog K. Distribution of cone density, spacing and arrangement in adult healthy retinas with adaptive optics flood illumination. PLoS One. 2018;13: e0191141. doi:10.1371/journal.pone.0191141

3.	Kim YJ, Joe SG, Lee D-H, Lee JY, Kim J-G, Yoon YH. Correlations between Spectral-Domain OCT Measurements and Visual Acuity in Cystoid Macular Edema Associated with Retinitis Pigmentosa. Investigative Ophthalmology & Visual Science. 2013;54: 1303–1309. doi:10.1167/iovs.12-10149

4.	Lammer J, Prager SG, Cheney MC, Ahmed A, Radwan SH, Burns SA, et al. Cone Photoreceptor Irregularity on Adaptive Optics Scanning Laser Ophthalmoscopy Correlates With Severity of Diabetic Retinopathy and Macular Edema. Invest Ophthalmol Vis Sci. 2016;57: 6624–6632. doi:10.1167/iovs.16-19537

5.	Akagi-Kurashige Y, Tsujikawa A, Ooto S, Makiyama Y, Muraoka Y, Kumagai K, et al. Retinal microstructural changes in eyes with resolved branch retinal vein occlusion: an adaptive optics scanning laser ophthalmoscopy study. Am J Ophthalmol. 2014;157: 1239-1249.e3. doi:10.1016/j.ajo.2014.02.026
