# Supplementary material for: Evaluation of photoreceptor features in retinitis pigmentosa with cystoid macular edema by using an adaptive optics fundus camera
Source: PLoS One. 2024 Jan 2;19(1):e0296493. doi: 10.1371/journal.pone.0296493 (PMC10760661; doi:10.1371/journal.pone.0296493)
Supplement: Attachment — Submitted filename: Response to Reviewers.docx [file pone.0296493.s006.docx]

Response to Reviewers

Evaluation of Photoreceptor Features in Retinitis Pigmentosa with Cystoid Macular Edema by Using an Adaptive Optics Fundus Camera (Manuscript #; PONE-D-23-24372)

All Concerned,

Thank you for inviting us to submit a revised manuscript. We appreciate the time and effort dedicated to providing insightful feedback to strengthen our manuscript. We have worked to address each concern by additional analysis and revising the text for clarity and added results. Thus, with great pleasure we submit our revised manuscript for further consideration. We have incorporated changes that reflect the detailed suggestions you have graciously provided. Hereafter we provide direct responses to each concern raised by the referees.

**Journal Requirements:**

**Please review your reference list to ensure that it is complete and correct. If you have cited papers that have been retracted, please include the rationale for doing so in the manuscript text, or remove these references and replace them with relevant current references. Any changes to the reference list should be mentioned in the rebuttal letter that accompanies your revised manuscript. If you need to cite a retracted article, indicate the article’s retracted status in the References list and also include a citation and full reference for the retraction notice.**

Thank you for pointing this out. We have reviewed the references again.

**Reviewer #1**

**The authors addressed the raised concerns. The comment AO imaging may detect photoreceptor loss that cannot be seen as EZ/IZ shortening is interesting, but it is not clearly stated in the manuscript. The authors may want to emphasize the significance of AO imaging.**

Thank you for pointing this out. We have added the following sentence to the main text. (Line 281)

**Reviewer #2: 1. Line 123 AO analysis; As pointed out in the first review, I am most concerned that the presence of edema itself will affect the AO camera imaging and cause the cone density to be measured lower. The authors state that “For each photoreceptor analysis examination, we captured the scans of the four perifoveal areas of the retina, as the regions of interest (ROI): superior, inferior, temporal, and nasal, 1.5° to 2° from the center of the fovea with a standardized sampling window of 62 × 62 μm sampling window size”. On the other hand, they also state that “In order to avoid the influence of CME on the measurement results of the AO examination, measurements were obtained from regions in which CME had occurred previously but not in areas with current edema in the OCT examination”.**

**⇒ Were not measurements taken at four fixed locations? If CME was present, did you exclude data from that measurement site? I recommend adding a typical example to Figure so that we can see how the measurements were taken in cases where CME is present.**

As you pointed out, the description was unclear and has been rewritten as following sentence. (Line128-132)

.

**2. The term "disease duration" or “duration” in the table 4 and in the text should be clearly stated as duration of CME, which is misleading as duration of retinitis pigmentosa.**

Thank you for your accurate point. There was indeed some confusion, so we have changed it to duration of CME.
